# Supplementary material for: MLND-IU: A multi-stage detection model of subcentimeter lung nodule with improved U-Net++
Source: PLoS One. 2026 Feb 6;21(2):e0341750. doi: 10.1371/journal.pone.0341750 (PMC12880664; doi:10.1371/journal.pone.0341750)
Supplement: Attachment — Submitted filename: Comments Dr. Muhammad Bilal Qureshi.pdf [file pone.0341750.s001.pdf]

MLND-IU: A Multi-stage Detection Model of Subcentimeter Lung Nodule with Improved U-Net++

The manuscript presents a multi-stage detection model with an improved U-Net++ for sub-centimeter lung tumor detection. The manuscript is well written and the presentation is also appropriate. The approach seems promising, particularly in reducing false positives and improving sensitivity through feature fusion. The topic is highly relevant to medical imaging and early detection of lung tumors. The attempt to enhance U-Net++ with multi-stage detection and feature fusion is innovative. The work claims improvements in false positive rate reduction and sensitivity.

However, there are several shortcomings that should be addressed before publication.

Technical Comments and mathematical Typos

1. The contributions are lengthy, repetitive, and not clearly distinguished.
2. Authors should rewrite them concisely (bullet-point style), clearly highlighting novelty, improvements over vanilla U-Net++/nnU-Net, and clinical impact.
3. The manuscript claims use of LIDC-IDRI and Kaggle DSB2017, which are appropriate. However, some text still refers to outdated datasets. Clarify and ensure all reported experiments are on recent, widely used benchmarks.
4. A clear **time-complexity analysis** of the three-stage pipeline (RetinaNet + AG-U-Net++ + 3D-CPM) is required.
5. Please report: FLOPs, parameter counts, memory usage, and latency per case (with hardware details).
6. Distinguish between full-volume and ROI-based segmentation. Compare runtime with baseline U-Net, U-Net++, and nnU-Net.
7. Evaluation is mostly Recall, Dice, and FP/Scan. Add **AUC, specificity, and precision** to strengthen evidence.
8. Comparisons should include **transformer-based approaches** (e.g., TransUNet, LN-DETR).
9. Ablation studies are good but should include statistical significance tests (confidence intervals).
10. **Eq. (22)** Recall incorrectly uses TN; correct formula is TP/(TP+FN)
11. **Eq. (23)** Dice coefficient incorrectly includes TN in denominator; correct form is 2TP/(2TP+FP+FN)

Language & Grammar (rephrasing required)

For example:

12. "Our multi stage detection model work with improved Unet++…"
13. "The contributions of this paper are given as follow but are not limited to…"
14. "We used the dataset from Kaggle which is freely available and popular for lung cancer detection from last many years."

15. "The proposed method shows improved results as compared to existing methods in sensitivity and false positive rate."
16. "Our multi stage detection model work with improved Unet++ for reducing false positive rate in subcentimeter tumor detection."

Figures & Tables

17. Improve figure captions, include what each figure demonstrates (e.g., highlight true vs. false positives).
18. Table 1 should clearly state dataset splits and preprocessing details.

References

19. Please add recent (2023–2025) transformer-based and hybrid attention models in medical imaging if possible.
