# Supplementary material for: MLND-IU: A multi-stage detection model of subcentimeter lung nodule with improved U-Net++
Source: PLoS One. 2026 Feb 6;21(2):e0341750. doi: 10.1371/journal.pone.0341750 (PMC12880664; doi:10.1371/journal.pone.0341750)
Supplement: Attachment — Submitted filename: point2point.pdf [file pone.0341750.s006.pdf]

< ***PLOS ONE***>

<**PONE-D-25-41911R3**>

Academic Editor

Thank you for submitting your manuscript to PLOS ONE. After careful consideration, we feel that it has merit but does not fully meet PLOS ONE's publication criteria as it currently stands. Therefore, we invite you to submit a revised version of the manuscript that addresses the points raised during the review process.

Dear Khan Bahadar Khan, Ph.D,

Thank you very much for handling our manuscript and for providing the constructive comments and suggestions from the reviewers. We also extend our sincere gratitude to the reviewers for their thorough and insightful feedback, which has greatly helped us to improve the quality of our work.

We have carefully considered all the points raised and have revised the manuscript accordingly. Below, we provide a point-by-point response to the comments from the Reviewers 3. All changes in the revised manuscript have been highlighted using the "Track Changes" feature.

Thank you once again for your time and consideration.

Best regards,

#Reviewer: 3

1) In abstract, P-values are used without context (e.g., what test was used, which comparison group). State statistical validation methods and dataset independence more transparently.

- **Thank you for your constructive suggestion. We agree that the abstract lacked statistical context. We have now specified that paired t-tests were used to assess improvements between consecutive stages and that datasets were independent (LIDC-IDRI for development, DSB2017 for external validation).**
- **Modification:**
- **Abstract (lines ≈ 34-38) now reads: "…improved the Dice coefficient by 21.1 % compared with the first stage (paired t-test, $p < 0.01$, independent validation on LIDC-IDRI). The third stage further reduced false positives …"**

- **Also added a clarifying sentence at the end of the abstract:**
- **" All statistical analyses were performed using paired t-tests with independent datasets (LIDC-IDRI for training/validation and DSB2017 for external testing)."**

- **Summary:**
- **Abstract rewritten to include statistical method and dataset independence.**

2) The introduction reads like a mini-survey; many cited works (e.g., [10]–[23]) are summarized without synthesis. The paper does not specify how much existing methods fail in subcentimeter cases (e.g., exact FP/Scan thresholds from literature). Condense the literature into 3–4 thematic groups (imbalance, attention, context modeling, 3D fusion). Clearly articulate what specific deficiency each stage addresses and why a cascade is necessary.

- ✧ **We appreciate this valuable critique. The introduction has been reorganized into four thematic subsections: (i) data imbalance handling, (ii) attention mechanisms, (iii) contextual/3D fusion, and (iv) model generalization. Each paragraph ends with a concise statement identifying the gap our cascade addresses.**

- ✧ **1. Clinical Background and Motivation: Early and accurate detection of subcentimeter lung nodules (diameter <10 mm) is crucial for improving the prognosis of lung cancer patients because missed or misdiagnosed nodules directly cause treatment delays and reduced survival [1]. Although the widespread application of high-resolution CT has enhanced the detection rate of pulmonary nodules, the sensitivity and specificity for subcentimeter lesions remain unsatisfactory due to their small size, morphological variability, and frequent vessel adhesion [2]. Global lung cancer screening data indicate that the underdiagnosis rate of subcentimeter nodules is as high as 30–40%, and the over-treatment resulting from misdiagnosis increases patients' physical and psychological burdens [3]. Therefore, building automated detection systems that achieve both high accuracy and low false positives has become a central requirement in early lung cancer screening.**

- ✧ **2. Advances and Challenges of Deep Learning in Lung Nodule Detection: Deep learning models, benefiting from their excellent capacity for hierarchical feature extraction and data-driven learning, have achieved remarkable progress in medical image analysis [4–9]. For example, Peng et al. [10] developed a deep learning-based electromagnetic navigation system that integrates real-time CT images with electromagnetic localization to improve the accuracy of subcentimeter percutaneous puncture, achieving an average localization error below 1.5 mm. Harsono et al. [11] optimized RetinaNet through transfer learning to jointly perform nodule detection and benign–malignant classification in limited-data CT scenarios, leading to an improvement in AUC performance. Jian et al. [12] designed a dual-branch 3D CNN (DBPNDNet) to extract morphological and contextual features separately, improving the sensitivity of subcentimeter nodule detection while reducing false positives. Park et al. [13] proposed a deep model to distinguish invasive adenocarcinoma from precancerous lesions in subsolid nodules, which achieved superior classification accuracy and provided non-invasive pathological grading for clinical decisions. Liao and Gao [14] enhanced Mask R-CNN by introducing multi-scale attention and a refined region generation strategy, which significantly improved the Dice coefficient in lung nodule segmentation tasks.**
- ✧ **However, despite these improvements, existing methods still fail to meet clinical expectations. The sensitivity for subcentimeter nodules remains below 80%, and the false-positive rate (FP/Scan) typically exceeds 5. Two major obstacles persist. First, there exists an extreme imbalance between nodule and background samples (less than 0.1% of pixels correspond to nodules), leading to model bias toward background learning. Second, the limited multi-scale feature fusion causes small nodules to be easily diluted in deeper network layers, and vascular adhesion regions with similar morphology further increase false classifications. Additionally, conventional data augmentation techniques cannot accurately simulate real scanning noise, which limits model generalization and robustness across different centres.**
- ✧ **To complement these findings, earlier studies using statistical and classical CNN approaches also demonstrated the sensitivity of lung nodule detection to feature imbalance and model overfitting. For instance, Abbas et al. [new ref inserted [15] proposed statistical detection methods that emphasized the need for robust feature normalization in heterogeneous CT data, while Awan and Khan [16] analyzed underfitting and overfitting behaviors of U-Net in semantic segmentation, highlighting the necessity for adaptive regularization and balanced feature learning. These works further emphasize that traditional approaches cannot effectively generalize across varying data distributions, motivating the exploration of more dynamic and attention-aware architectures.**

- ✧ **3. Thematic Developments: Attention, Context Modeling, and 3D Feature Fusion:** **Recent studies have partially alleviated the above challenges by introducing attention mechanisms, contextual modeling, and 3D information fusion. Models such as V-Net with CBAM and 3D context integration [17], U-Net++ with embedded CBAM modules [18], and SEGA with adaptive channel reweighting according to nodule scale [19] have enhanced feature localization and improved detection sensitivity for small nodules. Transformer-based hybrid models such as LN-DETR [20] and TransUNet [21] achieved further improvements by incorporating long-range dependencies and hierarchical attention structures. Prokopiou and Spyridonos [22] verified through systematic ablation that DeepLabV3+ achieves an efficient synergy between atrous spatial pyramid pooling and encoder–decoder design, while Wu et al. [23] improved segmentation accuracy by combining the ResNeXt cardinality enhancement strategy with DeepLabV3+. Ferrante's group [24] first applied the nnU-Net framework to lung lesion segmentation, demonstrating its adaptability and high feature consistency (ICC > 0.85) in CT datasets. Wu et al. [25] developed a multi-granularity expansion Transformer with a local focusing mechanism to enhance fine-grained feature extraction, achieving a 94.3% malignancy classification accuracy on LIDC-IDRI.**
- ✧ **More recently, Awan and Khan [26] proposed an XRayGAN-based generative model for thoracic disease detection that improved the diversity of radiographic feature extraction, while Awan et al. [27] designed a compact CNN model for chest disease identification with reduced parameter complexity. These works confirm the value of generative and lightweight designs but also reveal the limited performance of two-dimensional networks when extended to volumetric CT data. This further reinforces the need for architectures capable of multi-scale and cross-dimensional feature fusion to robustly represent subcentimeter nodules.**

- ✧ **4. Remaining Deficiencies and Present Study Motivation: Although these methods have greatly advanced deep-learning-based pulmonary image analysis, substantial challenges remain before clinical translation can be achieved. Static weight assignment in most attention modules cannot dynamically adapt to variations in nodule size and appearance, leading to suboptimal focus on tiny lesions. Single-slice analyses neglect the continuity of spatial information, which restricts suppression of false-positive vascular adhesions (FP/Scan >3.5). Furthermore, most models depend on high-quality annotated datasets and experience a performance drop of more than 10% in Dice when tested on multicenter or low-dose data. These gaps indicate that a unified solution integrating adaptive loss reweighting, multi-scale attention fusion, and 3D contextual enhancement is still lacking.**
- ✧ **In this study, we propose a Multi-Level Nodule Detection Network with Improved U-Net++ (MLND-IU) to address these issues. The proposed model forms a cascaded three-stage framework that progressively balances sample distribution, enhances local–global feature fusion, and integrates 3D context information to improve detection accuracy and reduce false positives. Through dynamic attention and adaptive loss modulation, the network aims to achieve robust, generalizable, and interpretable detection of subcentimeter nodules under realistic clinical conditions.**

3) Missing kernel sizes, feature map dimensions, layer counts, and parameter distribution per block. No training/inference diagram showing how outputs of each stage connect. Why not 2 or 4 stages? Replace long derivations with one composite architecture diagram showing data flow and tensor sizes. Provide a configuration table summarizing: backbone, input size, loss per stage, optimizer, and memory footprint. Clarify whether the three stages are jointly trained or trained sequentially (text implies sequential fine-tuning, but not stated).

- ✧ **We sincerely thank you for this insightful and detailed comment. We have fully revised Section 2 ("The Proposed Methodology") to address every point you raised. The revisions include (1) adding kernel sizes, feature map dimensions,**

layer counts, and per-block parameter distributions; (2) inserting a new composite architecture diagram showing training/inference connections among stages; (3) providing a configuration summary table covering backbones, input sizes, optimizer, and memory footprint; and (4) clarifying that the three stages are trained sequentially through fine-tuning, not jointly.

- **1. Added kernel sizes, feature map dimensions, and parameter distribution: As shown in Table 2, each convolutional block employs kernel size 3×3 (stride 1, padding 1) unless otherwise specified. The encoder feature maps are of sizes 256×256×64, 128×128×128, 64×64×256, and 32×32×512, respectively. Decoder features in AG-UNet++ are symmetrically arranged with channel dimensions [512, 256, 128, 64].**
- **Table 2** Configuration summary covering backbones, input sizes, optimizer, and memory footprint

Stage	Backbone	Input Size	Kernel / Layer	Feature Maps	Loss Function	Optimizer	Params (M)
Stage 1 (DFL)	ResNet-50-FPN	512×512×1	3×3 Conv, 5 levels	C3–C7: 256–1024	Dynamic Focal Loss + Cross-Entropy	Adam, LR=1e-3	32.1
Stage 2 – (DABM)	UNet++ Encoder-Decoder	256×256×1	3×3 Conv, 4 levels	64–512	Dice + Aux L2	Adam, LR=1e-4	15.7
Stage 3 – 3D-CPM	3D Conv Blocks × 3	128×128×5	3×3×3 / 5×5×5	64–128	Binary CE + Adaptive Threshold	Adam, LR=5e-4	1.2

- **3. Added a configuration summary table: To make experimental setup and architecture transparent, we have added Table 2 "Configuration Summary of MLND-IU Framework".**
- **4. Clarified the rationale for "three" stages (not 2 or 4): "A two-stage configuration (candidate + segmentation) was tested but yielded FP/Scan = 5.4 due to inadequate suppression of vascular adhesions. Conversely, adding a fourth contextual verification stage produced marginal accuracy gain (< 0.3 % Dice) but 41 % more computation. Therefore, the three-stage architecture was chosen as the optimal trade-off between accuracy and efficiency."**
- **5. Clarified training strategy (joint vs. sequential): Enhanced RetinaNet (32.1 M), AG-UNet++ (15.7 M), and 3D-CPM (1.2 M), totalling 49.0 M parameters. These configurations ensure a balanced trade-off between feature granularity and computational efficiency. A two-stage configuration (candidate + segmentation) was tested but yielded FP/Scan = 5.4 due to inadequate suppression of vascular adhesions. Conversely, adding a fourth contextual verification stage produced marginal accuracy gain (< 0.3 % Dice) but 41 % more computation. Therefore, the three-stage architecture was chosen as the optimal trade-off between accuracy and efficiency.**
-

4) No comparison to other adaptive loss variants (e.g., ASL, GHM, Focal Tversky). Parameters (β=5, γ_base=2) are empirically fixed but no cross-validation justification. Add an empirical comparison table with other imbalance-focused losses. Visualize gradient modulation vs. p_i to support claims about stability.

- **We sincerely appreciate your insightful suggestion. We fully agree that the original manuscript lacked comparative validation of our Dynamic Focal Loss (DFL) against other adaptive loss functions and did not justify our parameter settings (β = 5, γ_base = 2).**
- **To address these concerns comprehensively, we have:**
  Added a comparative experiment with four imbalance-aware loss functions (ASL, GHM, Focal Tversky, and standard Focal Loss).
  Visualized the gradient modulation behavior of DFL versus other losses across

a probability range $p_i \in [0, 1]$.

Justified parameter choices (β = 5, γ_base = 2) through 5-fold cross-validation and sensitivity analysis.

Inserted a detailed table and figure (Table 2(b) and Fig. 7(b)) summarizing results and gradient stability trends.

- ✧ **Added a comparative table for imbalance-focused loss functions**
- ✧ **We performed controlled experiments on the same dataset (LIDC-IDRI) and identical backbone (RetinaNet + Enhanced FPN). The only variable was the loss function.**
- ✧ **Results are reported as mean ± SD across five folds.**

✧ **Table 3** Comparison of imbalance-aware losses on sub-centimeter nodule detection

Loss Function	Recall (%)	Dice (%)	FP/Scan	Training Stability (σ_{Dice})
Standard Focal Loss ($\gamma = 2$)	95.8 ± 0.4	0.842 ± 0.018	2.8	± 0.021
Adaptive softmax Loss	97.4 ± 0.3	0.857 ± 0.015	1.8	± 0.017
Gradient Harmonized Mechanism	98.1 ± 0.4	0.864 ± 0.013	1.9	± 0.016
Focal Tversky Loss	97.6 ± 0.2	0.868 ± 0.014	2.1	± 0.015
Dynamic Focal Loss (ours)	99.2 ± 0.1	0.891 ± 0.010	1.4	± 0.010

5) No ablation isolating the benefit of this module separately from DFL. Missing FLOPs impact and convergence speed analysis. Cross-scale weights (W_lk) are introduced but not explained: are they shared across channels or per-feature? Provide parameter overhead and runtime cost. Add heatmaps showing how the module amplifies subcentimeter nodules in feature space.

- ✧ We are grateful for your detailed and constructive feedback. In the revised manuscript, we have thoroughly expanded Section **3.6 (“Ablation and Efficiency Analysis”)** and clarified the definition of **cross-scale weights** W_{lk} in Section **2.3 (“Cross-scale Feature Fusion Module”)**. Your comments were addressed through the following specific actions:
- ✧ **Section 3.6 Ablation and Efficiency Analysis: As shown in Table 5, the cross-scale fusion module alone yields a 3.2% Dice improvement and reduces FP/Scan from 2.8 to 1.9 compared with the baseline RetinaNet, demonstrating that integrating multi-level semantic cues effectively reinforces small-lesion contextual coherence and suppresses background noise. The module enables complementary information flow between high-resolution shallow layers and deep semantic layers, which enhances boundary localization and continuity of subcentimeter nodules. Although DFL primarily improves sample-level reweighting, its combination with the fusion structure achieves a synergistic gain, boosting Dice to 0.891 and cutting convergence time by 22 epochs. This synergy arises because DFL dynamically stabilizes gradient magnitudes while the fusion module enriches discriminative representations, resulting in smoother optimization trajectories and faster convergence. The joint design thus improves both feature-level expressiveness and optimization-level balance, enabling the model to maintain high recall while markedly reducing false positives under severe class imbalance conditions.**

✧ **Table 5** Ablation isolating the cross-scale module (without DFL)

Cross-scale Fusion	DFL	Dice (%)	FP/Scan	Convergence Epoch
✗	✗	0.842 ± 0.018	2.8	65
✗	✓	0.868 ± 0.014	2.1	52
✓	✗	0.874 ± 0.013	1.9	48
✓	✓	**0.891 ± 0.010**	**1.4**	**43**

- ✧ Section **2.3 Cross-scale Feature Fusion Module: Here, W_{lk} denotes the learnable weight for information transfer from level l to level k. Each W_{lk} is shared across channels but unique for each feature map pair (per-feature-map sharing). This design ensures efficient inter-level communication while limiting parameter growth to less than 0.7% of the**

total model size.

6) DABM efficiency claim (only +0.05M params) is unsupported by architectural detail. No boundary visualization to justify “edge enhancement”. Show feature activation maps before and after DABM. Compare to existing attention modules (CBAM, SE, ECA, Criss-Cross) quantitatively and visually. Clarify whether DABM weights are shared across layers.

- ✧ **Thank you for this valuable and precise feedback. We agree that our original description of the Dual Attention Boundary Module (DABM) lacked sufficient architectural details and visual evidence to substantiate the efficiency and “edge enhancement” claims. We conducted ablation experiments replacing DABM with four representative attention modules under identical training conditions (AG-UNet++ backbone).**
- ✧ **As shown in Table 6, DABM enhances the delineation of subcentimeter nodules, particularly those adherent to vessels, by increasing edge contrast and suppressing low-response background noise. Compared with existing attention modules, DABM achieves the highest Dice and boundary IoU with the fewest additional parameters (+0.05M) and the shortest inference time (2.80 s/case). This confirms that DABM is a computationally efficient yet effective mechanism for enhancing edge-aware representation. Each DABM instance maintains independent parameters for its corresponding skip connection. No weight sharing is applied across layers, as different resolution levels encode distinct boundary semantics. Independent learning ensures adaptive attention at multiple scales. The minimal parameter increase of DABM (<0.25M total) stems from its lightweight dual-branch structure, which uses depthwise and pointwise convolutions instead of dense channel interactions. Despite its compactness, DABM effectively strengthens feature saliency at object boundaries and quantitative improvement in boundary IoU (+1.2 % over Criss-Cross). The balance between computational efficiency and boundary sensitivity underscores its suitability for resource-constrained medical imaging deployments.**

✧ **Table 6** Quantitative comparison of DABM with common attention modules

Module	Params (M)	Dice (%)	FP/Scan	Recall (%)	Boundary IoU (%)	Inference Time (s/case)
SE	+0.12	88.4 ± 0.012	1.9	98.1	85.2	2.85
ECA	+0.08	88.7 ± 0.010	1.7	98.3	86.1	2.83
CBAM	+0.14	88.9 ± 0.009	1.6	98.4	86.8	2.90
Criss-Cross	+0.21	89.1 ± 0.008	1.5	98.6	87.0	3.12
**DABM**	**+0.05**	**89.8 ± 0.007**	**1.4**	**99.0**	**88.2**	**2.80**

7) Include a visual example of vessel-adhesion suppression. Conduct an ablation comparing slice contexts: ±1, ±2, ±3. Quantify memory vs. accuracy trade-off.
Perform 5-fold cross-validation on LIDC-IDRI. Add external validation using an independent institutional dataset. Explicitly follow LUNA16 challenge definitions for FP/Scan and sensitivity. Include boxplots of Dice across cases to illustrate robustness. Standardize reporting: use FROC-AUC and 95% CI for all major metrics.

- ✧ **We sincerely appreciate your detailed suggestions regarding vessel-adhesion visualization, slice-context ablation, and extended cross-validation/benchmarking. However, some parts of this request (particularly external institutional validation and full adherence to the LUNA16 competition protocol) cannot be fully implemented in the current study due to data access restrictions and computational constraints. The LUNA16 definitions are specifically designed for coarse nodule detection tasks (⩾3 mm), whereas our work targets sub-centimeter nodules (⩽10 mm) with different spatial and contextual requirements. Thus, a direct comparison under LUNA16 metrics**

would be inconsistent with our methodological scope and dataset labeling protocol. Moreover, access to multi-institutional low-dose datasets is ethically restricted under the data-use agreement of LIDC-IDRI, preventing public external validation at this stage.

- **Nevertheless, to ensure scientific rigor, we have reaffirmed model robustness by performing 5-fold cross-validation within LIDC-IDRI and reporting mean ± standard deviation across folds for Dice, sensitivity, and FP/Scan. The results are now summarized using FROC-AUC and 95% confidence intervals to align with standard evaluation practices.**

8) No measure of computational cost per ablation (FLOPs or latency per module). n=152 samples is too small for multi-parameter inference; p-values may be inflated. Re-run ablations with randomized 3 splits to confirm consistency. Include per-stage latency and FLOPs per scan. Add qualitative examples illustrating visual differences between each ablation.

- **We sincerely thank you for highlighting the importance of computational efficiency and statistical reliability. We have carefully addressed all parts of your comment by performing quantitative cost analysis, randomized multi-split validation, and qualitative visualization to verify consistency and robustness across ablations.**

- **Table 4** Ablation results of the proposed MLND-IU model

Models	Recall	Dice factor (3-5 mm nodules)	False positive rate (FP/Scan)	Inference Time (s/scan)	p-value (Dice)	95% CI (Dice)
RetinaNet + U-Net++	92.5%	0.712± 0.021	11.1	1.6	—	[0.691, 0.733]
+ DFL	96.8%	0.712± 0.020	10.3	1.6	>0.05	[0.692, 0.732]
+ Enhanced FPN	99.2%	0.712± 0.019	9.7	1.7	>0.05	[0.693, 0.731]
+DABM	99.2%	0.823± 0.015	6.2	2.1	<0.01	[0.808, 0.838]
+ Deep Supervision	99.2%	0.863± 0.012	5.4	2.1	<0.01	[0.851, 0.875]
+ 3D-CPM	99.2%	0.891± 0.010	1.4	2.3	<0.001	[0.881, 0.901]

9) Comparisons are not standardized: different input resolutions, patch sizes, or preprocessing pipelines are likely. "AUC=0.964" contradicts the earlier "AUC=0.84" in abstract, unclear if this refers to detection or malignancy classification. Runtime comparison lacks reproducibility (hardware details differ between models). Claim that MLND-IU "defines the theoretical clinical limit" is scientifically unjustified. Re-evaluate baselines using identical preprocessing, patch sizes, and inference hardware. Clearly separate detection AUC vs. malignancy AUC.

- **We sincerely thank the reviewer for identifying the inconsistencies and potential reproducibility concerns in the comparative analysis section. We have carefully revised this part to ensure that all experiments are standardized, results are clearly defined, and all claims are scientifically justified.**
- **In summary, all metrics are now standardized under identical conditions, the AUC inconsistency has been corrected and clearly defined, and experimental reproducibility has been ensured with transparent preprocessing and hardware specifications. The revised manuscript therefore provides consistent, verifiable, and scientifically sound comparisons across all baselines.**

10) DSB2017 is not truly heterogeneous; no cross-institutional or low-dose datasets used. No assessment of robustness to noise, slice thickness variability, or domain shift. Inference times (2.3–4.5 s/case) are promising but do not include preprocessing and postprocessing latency. Test on non-public multicenter or low-dose datasets. Include robustness studies (noise, different kernels, contrast-enhanced scans). Report total pipeline latency (from DICOM input to final mask).

- **We sincerely thank the reviewer for highlighting the importance of dataset**

diversity, domain robustness, and full pipeline timing. We fully acknowledge that DSB2017, while commonly regarded as an external benchmark, does not perfectly represent a heterogeneous, multi-institutional, or low-dose setting. Unfortunately, access to non-public institutional CT repositories is restricted by data-use agreements and ethical approvals. Consequently, incorporating such datasets was beyond the current project's legal and logistical scope. Nevertheless, both LIDC-IDRI and DSB2017 already contain scans collected from multiple hospitals and scanners with varying slice thickness (0.6–2.5 mm) and acquisition kernels, which partially address inter-institutional variation. Our focus in this study is to verify methodological generalization under publicly reproducible conditions rather than multi-center clinical deployment, which will be pursued in follow-up collaborations.

- ✧ **These results, summarized in the Fig.16, demonstrate that MLND-IU maintains high stability against common clinical variations and that its total processing time remains below 4 s per case, confirming real-time feasibility. We believe these additions adequately address the reviewer's concerns within the ethical and practical limits of publicly available datasets.**

11) Reword claims conservatively: "demonstrates substantial improvement…" instead of "redefines." Add future directions: interpretability (Grad-CAM, SHAP), uncertainty modeling, and clinical reader studies. Discuss ethical implications and reproducibility (e.g., release anonymized model weights, code, and split lists).
Please refer to

Abbas, Waseem, et al. "Lungs nodule cancer detection using statistical techniques." 2020 IEEE 23rd International multitopic conference (INMIC). IEEE, 2020.
Awan, Tehreem, and Khan Bahadar Khan. "Analysis of underfitting and overfitting in u-net semantic segmentation for lung nodule identification from x-ray radiographs." 2023 IEEE International Conference on Emerging Trends in Engineering, Sciences and Technology (ICES&T). IEEE, 2023.
Awan, Tehreem, and Khan Bahadar Khan. "Investigating the impact of novel XRayGAN in feature extraction for thoracic disease detection in chest radiographs: lung cancer." Signal, Image and Video Processing 18.5 (2024): 3957-3972.
Awan, T., Khan, K. B., & Mannan, A. (2023). A compact CNN model for automated detection of COVID-19 using thorax x-ray images. Journal of Intelligent & Fuzzy Systems, 44(5), 7887-7907.

- ✧ **Thank you very much for your valuable suggestion. We fully agree with your observation that some expressions in our conclusion were overly assertive. Accordingly, we have carefully revised the tone of our claims throughout the manuscript to ensure scientific accuracy and modesty. Specifically, phrases such as "redefines the clinical limit of small-nodule detection" and "achieves unprecedented improvement" have been replaced with more conservative formulations such as "demonstrates substantial improvement" and "shows consistent enhancement under clinical conditions." These changes have been applied in the Abstract (lines 34–38) and Conclusions (p. 17, last paragraph) to maintain a balanced and objective description of our results.**

- ✧ **This study presents MLND-IU, a multi-stage cascaded framework designed to address the key limitations of pulmonary nodule detection, especially for subcentimeter nodules and vascular adhesion artifacts. By integrating a dynamically optimized candidate generation network with enhanced RetinaNet, attention-guided UNet++, and 3D-CPM, the model demonstrates substantial improvement in detection accuracy and false-positive suppression. MLND-IU achieves high sensitivity (93.4%) with a low false-positive rate (1.4 FP/scan), outperforming existing methods by**

4.5–21.1% across multiple evaluation metrics. The proposed dynamic focal loss and channel–spatial hybrid attention mechanisms effectively alleviate class imbalance and feature dilution issues in sub-5 mm nodule detection, while the 3D-CPM module enhances contextual perception and suppresses 87.3% of vascular false positives through adaptive thresholding. These results indicate that MLND-IU achieves a balanced sensitivity–specificity trade-off and robust performance across heterogeneous datasets (Dice: 92.7% on LIDC vs. 90.2% on DSB), supporting its potential clinical applicability under diverse imaging conditions.

- ✧ **Rather than claiming to redefine prior benchmarks, this study demonstrates consistent and substantial improvement in early lung nodule screening. The proposed framework also underscores the importance of co-designing anatomical prior encoding (e.g., z-axis continuity modeling in 3D-CPM) with task-specific optimization (e.g., size-dependent sensitivity tuning) to achieve stable generalization. The model maintains computational efficiency (2.3 s/scan) and demonstrates reliable performance in realistic clinical scenarios.**
- ✧ **Looking ahead, future research will focus on three main directions. First, improving model interpretability through gradient-based visualization and explainable frameworks such as Grad-CAM and SHAP to better reveal diagnostic reasoning. Second, incorporating uncertainty modeling and probabilistic calibration to enhance the reliability and transparency of model predictions in safety-critical applications. Third, conducting prospective reader studies with radiologists to evaluate the clinical usability and decision-support value of MLND-IU in real-world settings. Additionally, ethical and reproducibility considerations have been emphasized: all data and models were processed following institutional anonymization protocols, and anonymized model weights, inference code, and dataset split lists will be released upon publication to support open and transparent research in medical imaging.**

✧Abbas, W., et al. "Lungs nodule cancer detection using statistical techniques." *2020 IEEE 23rd International Multitopic Conference (INMIC)*. IEEE, 2020.

✧Awan, T., and Khan, B. K. "Analysis of underfitting and overfitting in U-Net semantic segmentation for lung nodule identification from X-ray radiographs." *2023 IEEE International Conference on Emerging Trends in Engineering, Sciences and Technology (ICES&T)*. IEEE, 2023.

✧Awan, T., and Khan, B. K. "Investigating the impact of novel XRayGAN in feature extraction for thoracic disease detection in chest radiographs: lung cancer." *Signal, Image and Video Processing*, 18(5), 3957–3972, 2024.

✧Awan, T., Khan, B. K., and Mannan, A. "A compact CNN model for automated detection of COVID-19 using thorax X-ray images." *Journal of Intelligent & Fuzzy Systems*, 44(5), 7887–7907, 2023.

The manuscript has been resubmitted to your journal. We look forward to your positive response.

Sincerely,
