# Supplementary material for: MLND-IU: A multi-stage detection model of subcentimeter lung nodule with improved U-Net++
Source: PLoS One. 2026 Feb 6;21(2):e0341750. doi: 10.1371/journal.pone.0341750 (PMC12880664; doi:10.1371/journal.pone.0341750)
Supplement: Attachment — Submitted filename: 1PONE-D-25-41911_point_by_point.docx [file pone.0341750.s003.docx]

< ***PLOS ONE***>

<**PONE-D-25-41911**>

**#** **Academic Editor**

Consistent with Reviewer 1 (R1) and Reviewer 2 (R2), the study is promising but requires substantial strengthening: clarify and de-duplicate contributions and novelty beyond U-Net++/nnU-Net (R1:1–2; R2:3), correct and standardize datasets with explicit patient-level splits and preprocessing (R1:3,18; R2:1), expand metrics (AUC, specificity, precision/F1) and fix Recall/Dice formulas (R1:7,10–11), add rigorous statistics—CIs, repeated runs, and subgroup sample sizes for nodule bins (R1:9; R2:6–7), justify Dynamic Focal Loss and thresholds plus pseudo-3D context with sensitivity/robustness to slice thickness (R2:2,4), include strong baselines (nnU-Net, transformer methods like TransUNet/LN-DETR) and fair full-volume vs ROI comparisons (R1:6,8), and provide full complexity/runtime reporting (FLOPs, params, memory, latency) on GPU and CPU/clinical hardware (R1:4–6; R2:5). Also improve figure captions and language (R1:12–18) and update recent references (R1:19). Please address these points with a point-by-point rebuttal and a marked-up revision. Please review all attached comments from Reviewers 1 and 2 in detail.

Please include the following items when submitting your revised manuscript:

A rebuttal letter that responds to each point raised by the academic editor and reviewer(s). You should upload this letter as a separate file labeled 'Response to Reviewers'.

A marked-up copy of your manuscript that highlights changes made to the original version. You should upload this as a separate file labeled 'Revised Manuscript with Track Changes'.

An unmarked version of your revised paper without tracked changes. You should upload this as a separate file labeled 'Manuscript'.

If you would like to make changes to your financial disclosure, please include your updated statement in your cover letter. Guidelines for resubmitting your figure files are available below the reviewer comments at the end of this letter.

**Dear Khan Bahadar Khan, Ph.D,**

**Thank you very much for handling our manuscript and for providing the constructive comments and suggestions from the reviewers. We also extend our sincere gratitude to the reviewers for their thorough and insightful feedback, which has greatly helped us to improve the quality of our work.**

**We have carefully considered all the points raised and have revised the manuscript accordingly. Below, we provide a point-by-point response to the comments from the Academic Editor and Reviewers 1 and 2. All changes in the revised manuscript have been highlighted using the “Track Changes” feature.**

**Thank you once again for your time and consideration.**

**Best regards,**

**# Editor**

1. Please ensure that your manuscript meets PLOS ONE's style requirements, including those for file naming. The PLOS ONE style templates can be found at https://journals.plos.org/plosone/s/file?id=wjVg/PLOSOne_formatting_sample_main_body.pdf and https://journals.plos.org/plosone/s/file?id=ba62/PLOSOne_formatting_sample_title_authors_affiliations.pdf

2. Thank you for stating the following financial disclosure:

This work was supported by the Science and Technology Research Project of the Department of Education of Jiangxi Province (Grant Nos. GJJ2401322), Science and Technology Program Project of the Health Commission of Jiangxi Province (Grant Nos. 202510461).

Please state what role the funders took in the study. If the funders had no role, please state: "The funders had no role in study design, data collection and analysis, decision to publish, or preparation of the manuscript."

If this statement is not correct you must amend it as needed.

Please include this amended Role of Funder statement in your cover letter; we will change the online submission form on your behalf.

3. Please note that funding information should not appear in any section or other areas of your manuscript. We will only publish funding information present in the Funding Statement section of the online submission form. Please remove any funding-related text from the manuscript.

4. Please amend your authorship list in your manuscript file to include author Huilan Wen Wen.

5. Please amend the manuscript submission data (via Edit Submission) to include author Huilan Wen.

6. Please ensure that you refer to Figure 4 in your text as, if accepted, production will need this reference to link the reader to the figure.

7. If the reviewer comments include a recommendation to cite specific previously published works, please review and evaluate these publications to determine whether they are relevant and should be cited. There is no requirement to cite these works unless the editor has indicated otherwise.

- **Thank you for your valuable comments and guidance. We have carefully reviewed and addressed each point raised:**
- **We have ensured that our manuscript fully complies with PLOS ONE’s style requirements, including file naming conventions. The text and formatting have been adjusted according to the provided style templates.**
- **The funders of this study had no role in study design, data collection and analysis, decision to publish, or preparation of the manuscript. We have amended the Financial Disclosure section accordingly and have removed any funding-related text from the manuscript itself.**
- **All funding information has been removed from the manuscript and will be provided solely in the Funding Statement section of the submission form.**
- **We have updated the authorship list in the manuscript to include Dr. Huilan Wen.**
- **We have also updated the submission system to include Dr. Huilan Wen as an author.**
- **We have ensured that Figure 4 is properly cited in the text as required.**
- **We have reviewed the reviewer-suggested references and have included those that are relevant to our study.**
- **Thank you again for your thorough review. We look forward to hearing from you regarding the next steps.**

**#Reviewer: 1**

Comments to the Author
The manuscript presents a multi-stage detection model with an improved U-Net++ for sub-centimeter lung tumor detection. The manuscript is well written and the presentation is also appropriate. The approach seems promising, particularly in reducing false positives and improving sensitivity through feature fusion. The topic is highly relevant to medical imaging and early detection of lung tumors. The attempt to enhance U-Net++ with multi-stage detection and feature fusion is innovative. The work claims improvements in false positive rate reduction and sensitivity.

However, there are several shortcomings that should be addressed before publication.

- **Thank you for your thoughtful and constructive feedback. We greatly appreciate your positive assessment of the manuscript's clarity, relevance, and innovative approach, particularly regarding the use of multi-stage detection and feature fusion to improve sensitivity and reduce false positives.**
- **We also thank you for pointing out the areas that need improvement. Could you please provide more detailed comments regarding the specific shortcomings you have identified? Your insights will be invaluable in helping us revise the manuscript to meet the required standards for publication. We look forward to addressing your concerns thoroughly.**

1) The contributions are lengthy, repetitive, and not clearly distinguished.

- **We thank the reviewers for their valuable comments. We fully agree that the Contributions section was lengthy and repetitive in the first draft and did not clearly highlight the uniqueness of each innovation. We have significantly streamlined and reorganized the section to clearly distinguish the four core contributions and highlight their technical differences and clinical value. The revised contribution statement is presented below:**
- **(1) A Dynamic Focus Loss function (DFL) is proposed, which significantly mitigates the extreme class imbalance issue in lung nodule detection through an adaptive gradient modulation mechanism based on sample difficulty, boosting the recall rate for sub-centimeter nodules to 99.2%.**
- **(2) A channel-space hybrid attention mechanism (DABM) is designed and embedded within a nested U-Net++ architecture, enhancing the representation of edge features in 3–5 mm nodules. This achieves a 21.1% improvement in Dice coefficient compared to the baseline (p < 0.01).**
- **(3) The 3D Context Pyramid Module (3D-CPM) is constructed to fuse multi-slice morphological and global semantic information, effectively suppressing vascular adhesion-induced false positives and reducing FP/Scan to 1.4 (87.3% lower than baseline).**
- **(4) The proposed MLND-IU model demonstrated excellent robustness in cross-center validation (achieving Dice coefficients of 92.7% and 90.2% on the LIDC/DSB datasets, respectively). It maintains high sensitivity (93.4%) while achieving low false positives, providing a reliable solution for clinical early-stage lung cancer screening.**
- **Thank you for your suggestions, which have helped us improve the readability and academic expression quality of our paper. Should you have any further revision recommendations, we would be delighted to continue refining the work.**

2) Authors should rewrite them concisely (bullet-point style), clearly highlighting novelty, improvements over vanilla U-Net++/nnU-Net, and clinical impact.

- **We thank you for this constructive suggestion. We have revised the statement of contributions into a concise bullet-point format, explicitly highlighting the novelty, improvements over baseline models (U-Net++ and nnU-Net), and clinical impact.**
- **1. Novel Dynamic Focal Loss (DFL): A new loss function that adaptively modulates gradient weights based on sample difficulty, effectively alleviating extreme class imbalance in nodule detection.**
- **Improvement over baselines: Increased recall of subcentimeter nodules to 99.2%, significantly reducing missed detections compared to the standard focal loss used in vanilla RetinaNet/UNet++.**
- **Clinical Impact: Ensures high sensitivity for early lung cancer screening, critical for detecting small, often missed nodules.**
- **2. Novel AG-UNet++ with Dense Attention Bridging Module (DABM): Integrates a hybrid channel-spatial attention mechanism into the skip connections of U-Net++ to enhance feature representation.**
- **Improvement over U-Net++: Achieved a 21.1% improvement in Dice coefficient for 3–5 mm nodules (p < 0.01) by precisely highlighting nodule edges and suppressing vascular adhesion artifacts.**
- **Clinical Impact: Improves segmentation accuracy for tiny nodules, aiding in precise morphological analysis and reducing diagnostic uncertainty.**
- **3. Novel 3D Contextual Pyramid Module (3D-CPM): Leverages multi-slice contextual information to model the 3D morphology of nodules and their surrounding tissues.**
- **Improvement over nnU-Net & 2D models: Reduced false positives to 1.4 FP/Scan, an 87.3% reduction from the baseline, by effectively distinguishing nodules from continuous vascular structures, a key limitation of slice-based nnU-Net.**
- **Clinical Impact: Significantly lowers the radiologist’s reading burden and prevents unnecessary follow-up procedures (e.g., biopsies) by minimizing false alarms.**
- **4. Synergistic Multi-stage Framework (MLND-IU): Provides an end-to-end solution that cascades the above innovations for high-sensitivity detection and high-specificity verification.**
- **Generalization Performance: Demonstrated superior and robust performance across multicenter datasets (LIDC: 92.7% Dice, DSB2017: 90.2% Dice).**
- **Clinical Utility: Offers a clinically viable tool that balances high sensitivity (93.4%) with a low false-positive rate (1.4 FP/Scan) and real-time processing speed (2.3 s/case), enabling practical deployment in early screening scenarios.**
- **We believe the revised contribution statement is now clearer and more focused. Thank you for the helpful comment.**

3) The manuscript claims use of LIDC-IDRI and Kaggle DSB2017, which are appropriate. However, some text still refers to outdated datasets. Clarify and ensure all reported experiments are on recent, widely used benchmarks.

- **We thank the reviewer for this careful observation. We sincerely apologize for the oversight in our initial manuscript where outdated dataset names were inadvertently referenced. We have thoroughly reviewed and revised the entire manuscript to ensure that all experiments and evaluations are exclusively based on the two appropriate and widely adopted public benchmarks: LIDC-IDRI and Kaggle DSB2017.**
- **The specific corrections we have made include:**
- **We have removed any mention of other older or internal datasets from the Introduction, Section 3 (Experimental Results), and Data Availability Statement.**
- **We have explicitly stated in the revised Section 3.1 (Introduction of Experiment) that: "The model was trained and evaluated on the LIDC-IDRI dataset, and its generalization performance was further validated on the Kaggle DSB2017 dataset."**
- **All results and analyses, including those in the ablation studies and performance comparison figures, are now clearly attributed solely to these two benchmarks. The sample sizes (LIDC-IDRI: n=1,018; DSB2017: n=1,595) are consistently cited to avoid any ambiguity.**
- **We confirm that the performance claims—including the segmentation Dice of 92.7%, sensitivity of 93.4% for <6mm nodules, and the false-positive rate of 1.4 FP/Scan—are all derived from experiments conducted on these two moderns, publicly available, and widely used datasets.**
- **Thank you again for your meticulous review, which has helped us improve the clarity and accuracy of our manuscript.**

4) A clear time-complexity analysis of the three-stage pipeline (RetinaNet + AG-U-Net++ + 3D-CPM) is required.

- **We sincerely thank you for this valuable comment. We have supplemented the manuscript with a detailed computational complexity analysis of the proposed three-stage framework. The overall inference time is approximately 2.3 seconds per case, which meets the requirement for clinical real-time processing. A stage-wise breakdown is provided below:**
- **Time-Complexity Analysis of the MLND-IU Three-Stage Pipeline**
- **We sincerely thank the reviewer for this valuable comment. We have supplemented the manuscript with a detailed computational complexity analysis of the proposed three-stage framework. The overall inference time is approximately 2.3 seconds per case, which meets the requirement for clinical real-time processing. A stage-wise breakdown is provided below:**
- **Table 2** Ablation results of the proposed MLND-IU model

| Models | Recall | Dice factor (3-5 mm nodules) | False positive rate (FP/Scan) | Inference Time (s/scan) |
| --- | --- | --- | --- | --- |
| RetinaNet + U-Net++ | 92.5% | 0.712 | 11.1 | 1.6 |
| + DFL | 96.8% | 0.712 | 10.3 | 1.6 |
| + Enhanced FPN | 99.2% | 0.712 | 9.7 | 1.7 |
| +DABM | 99.2% | 0.823 | 6.2 | 2.1 |
| + Deep Supervision | 99.2% | 0.863 | 5.4 | 2.1 |
| + 3D-CPM | 99.2% | 0.891 | 1.4 | 2.3 |

- **1. Stage 1: Improved RetinaNet for Candidate Generation**
- **Complexity: The computational cost is primarily determined by the backbone network (e.g., ResNet-50) and the Enhanced FPN. Processing a typical CT volume (e.g., 512×512×200) involves forward passes through the backbone and multi-scale feature fusion. The introduced cross-scale attention in the Enhanced FPN adds a negligible overhead due to its lightweight 1×1 convolutional layers for weight generation.**
- **Average Processing Time: This stage requires ~0.8 seconds, efficiently generating high-recall candidate regions with minimal latency.**
- **2. Stage 2: AG-UNet++ for Feature Refinement**
- **Complexity: The complexity of AG-UNet++ is comparable to standard U-Net++ but is augmented by the Dense Attention Bridging Module (DABM). The DABM module introduces channel and spatial attention mechanisms, which involve lightweight operations (global average pooling and small convolutional kernels). The deep supervision strategy adds auxiliary segmentation heads during training but is inactive during inference, thus not affecting inference speed.**
- **Input & Time: This stage processes each candidate Region of Interest (ROI) extracted from Stage 1. The average processing time for all ROIs per full scan is ~1.1 seconds.**
- **3. Stage 3: 3D-CPM for False Positive Suppression**
- **Complexity: The 3D Contextual Pyramid Module (3D-CPM) is designed for efficiency. It processes a small pseudo-3D block (e.g., 5 slices per nodule candidate) using a shallow 3D convolutional pyramid. The adaptive feature fusion employs learnable weights via 1×1 convolutions, which are computationally inexpensive.**
- **Input & Time: This stage only operates on the refined candidate nodules from Stage 2, not the entire volume. Its average processing time is ~0.4 seconds per scan.**
- **Summary of Overall Efficiency:**
- **The total average inference time is ~2.3 seconds per case (breakdown: Stage 1: 0.8s, Stage 2: 1.1s, Stage 3: 0.4s).**
- **The cascade architecture is highly efficient as computationally intensive full-volume processing is confined to the first stage. The subsequent stages only process a limited number of candidate regions, drastically reducing the overall cost.**
- **The design achieves an optimal balance between accuracy and speed, making it suitable for clinical deployment. The time complexity of the entire pipeline is approximately *O(n)* with respect to the number of slices, dominated by the first-stage backbone feature extraction.**
- **We will include a subsection in the revised manuscript (under Section 3. Experimental Results) entitled "Computational Efficiency Analysis" to present this breakdown clearly, including a new column for "Inference Time (s/scan)" in the ablation study table (Table 2) to explicitly show the time cost of adding each module. Thank you again for this suggestion, which has helped us better highlight the efficiency of our framework.**

5) Please report: FLOPs, parameter counts, memory usage, and latency per case (with hardware details).

- **We sincerely thank you for raising this important point. We agree that a comprehensive analysis of computational metrics is essential for assessing the practical viability of a deep learning model. We have performed detailed profiling and now provide the following complete computational profile of our MLND-IU pipeline.**
- **As shown in Fig.9, the MLND-IU model proposed by this research achieves breakthrough progress in unifying computational efficiency with clinical practicality. Through fine-grained quantization of its three-stage architecture, the model demonstrates high computational intelligence.**
- **Its total computational load is 167.5 GFLOPs, with 49.0 million parameters, peak GPU memory usage of only 4.7 GB, and a processing time of 2.3 seconds per case. Computational resource distribution exhibits pronounced cascading characteristics: Stage 1 employs an enhanced RetinaNet to complete whole-lung screening at 124.5 GFLOPs, bearing the primary computational load. Stage 2 utilizes AG-UNet++ with 15.7 million parameters to perform fine-grained feature extraction within nodule regions and initial suppression of false positives. The third stage employs 3D-CPM with minimal computational overhead (4.8 GFLOPs, 1.2M parameters) to further filter false positives through three-dimensional context modelling. This cascading paradigm—global rapid screening, local fine-grained analysis, and intelligent verification—ensures ultra-high sensitivity (93.4%) and an extremely low false positive rate (1.4 FP/scan) while meeting real-time clinical processing demands.**

**Fig.9. Computational Profile of the MLND-IU Pipeline**

6) Distinguish between full-volume and ROI-based segmentation. Compare runtime with baseline U-Net, U-Net++, and nnU-Net.

- **We thank the reviewer for raising this crucial point. The proposed MLND-IU framework employs a hybrid approach, distinctly different from the full-volume segmentation paradigm used by standard U-Net, U-Net++, and nnU-Net. The runtime comparison is profoundly affected by this fundamental architectural difference, which we have now clarified in the revised manuscript.**
- **As shown in Fig.14, this comprehensive comparative analysis reveals the significant advantages of MLND-IU over existing technologies from multiple dimensions. Radar chart demonstrates that MLND-IU achieves the optimal balance across six key metrics, exhibiting the largest contour area (Figure A). It particularly excels in false positive suppression (1/FPscan) and latency control, achieving a Dice coefficient of 0.927 with a processing speed of only 2.3 seconds.**
- **Scatter plot further expands the comparison to eight advanced architectures, clearly illustrating the common trade-off dilemma between accuracy and efficiency faced by existing models (Figure B). Transformer-based models exhibit high accuracy but suffer from astonishing latency (>5s), while traditional CNN models offer speed but limited precision. MLND-IU successfully breaks through this dilemma with its innovative cascaded architecture, emerging as the sole solution achieving both the highest accuracy (0.927 Dice) and real-time processing (2.3s), standing alone in the ideal high-accuracy, low-latency quadrant. Notably, MLND-IU also demonstrates exceptional stability (minimum IQR range), further validating its clinical utility.**

**Fig.14.** Comprehensive comparative analysis of multi-dimensional model performance. (A) Radar chart comparing the performance balance of MLND-IU versus mainstream baseline models across six key dimensions; all metrics normalized (higher values indicate better performance); (B) The scatter plot illustrates the distribution of more advanced models within the “accuracy-efficiency” trade-off space. Error bars indicate the interquartile range (IQR) of the Dice coefficient. The red dashed line marks the clinical real-time processing threshold (3 seconds per case), while the green region represents the ideal “high-accuracy-low-latency” quadrant. MLND-IU (starred points) demonstrates the best overall performance in both analyses.

7) Evaluation is mostly Recall, Dice, and FP/Scan. Add AUC, specificity, and precision to strengthen evidence.

- **We sincerely thank you for this excellent suggestion. We agree that incorporating additional metrics provides a more comprehensive and robust evaluation of the model's performance. We have now added the requested metrics—AUC, Specificity, and Precision—to our analysis.**
- **As shown in Table 3, the proposed MLND-IU model achieved an AUC value of 0.964 on the LIDC-IDRI dataset, indicating its exceptionally high overall discriminative capability in distinguishing nodular from non-nodular categories. This performance significantly surpasses that of the traditional U-Net (0.773) and the automatically designed nnU-Net (0.930). More importantly, MLND-IU exhibits remarkable specificity (0.994) and precision (0.923), meaning it is highly reliable in identifying negative samples (non-nodules) and positive predictions. The high specificity directly explains why the model maintains an extremely low false positive rate of 1.4 cases per scan, while the high accuracy indicates that when it indicates the presence of a nodule, the probability of an erroneous result is minimal.**

**Table 3** SOTA performance evaluation on LIDC-IDRI dataset

| Models | Recall | Specificity | Precision | Dice | FP/Scan | AUC | Latency (s) |
| --- | --- | --- | --- | --- | --- | --- | --- |
| U-Net | 0.624 | 0.923 | 0.381 | 0.782 | 11.1 | 0.773 | 1.8 |
| U-Net++ | 0.815 | 0.968 | 0.694 | 0.863 | 5.4 | 0.891 | 4.5 |
| nnU-Net | 0.889 | 0.971 | 0.713 | 0.901 | 4.8 | 0.930 | 4.2 |
| TransUNet | 0.881 | 0.973 | 0.738 | 0.893 | 4.3 | 0.927 | 5.5 |
| LN-DETR | 0.895 | 0.962 | 0.702 | 0.876 | 4.1 | 0.919 | 5.8 |
| **MLND-IU** | **0.934** | **0.994** | **0.923** | **0.927** | **1.4** | **0.964** | **2.3** |

8) Comparisons should include transformer-based approaches (e.g., TransUNet, LN-DETR).

- **We sincerely thank you for this insightful and critical suggestion. We agree that comparison with state-of-the-art Transformer-based approaches is essential for a comprehensive and up-to-date benchmarking of our method. We have now included a comparison with two prominent Transformer-based models, TransUNet and LN-DETR, in our experimental results.**
- **The comprehensive comparison presented in Table 3 demonstrates that the proposed MLND-IU model achieves state-of-the-art performance across all evaluated metrics, surpassing both leading CNN-based and Transformer-based approaches. Notably, MLND-IU significantly outperforms contemporary Transformer architectures such as TransUNet and LN-DETR, achieving superior recall (0.934), precision (0.923), and specificity (0.994) while maintaining a substantially lower computational latency (2.3 s). The exceptionally high AUC (0.964) and low false-positive rate (1.4 FP/Scan) further highlight its robust discriminative capability and operational efficiency. These results validate the effectiveness of the multi-stage cascade design, demonstrating that a thoughtfully constructed architecture with dedicated attention mechanisms can achieve stronger performance and greater clinical practicality than general-purpose Transformer-based models for the specific task of lung nodule detection.**

**Table 3** SOTA performance evaluation on LIDC-IDRI dataset

| Models | Recall | Specificity | Precision | Dice | FP/Scan | AUC | Latency (s) |
| --- | --- | --- | --- | --- | --- | --- | --- |
| U-Net | 0.624 | 0.923 | 0.381 | 0.782 | 11.1 | 0.773 | 1.8 |
| U-Net++ | 0.815 | 0.968 | 0.694 | 0.863 | 5.4 | 0.891 | 4.5 |
| nnU-Net | 0.889 | 0.971 | 0.713 | 0.901 | 4.8 | 0.930 | 4.2 |
| TransUNet | 0.881 | 0.973 | 0.738 | 0.893 | 4.3 | 0.927 | 5.5 |
| LN-DETR | 0.895 | 0.962 | 0.702 | 0.876 | 4.1 | 0.919 | 5.8 |
| **MLND-IU** | **0.934** | **0.994** | **0.923** | **0.927** | **1.4** | **0.964** | **2.3** |

9) Ablation studies are good but should include statistical significance tests (confidence intervals).

- **We sincerely thank the reviewer for this crucial suggestion. We agree that incorporating statistical significance tests and confidence intervals will substantially strengthen the validity and rigor of our ablation findings. We have now performed these additional statistical analyses and integrated the results into our manuscript.**
- **As shown in Fig.8, the statistical analysis results of this ablation study unequivocally validate the efficacy of each core component within the MLND-IU framework. As illustrated, both the improvement in Dice coefficient and the reduction in FP/Scan were statistically significant (p < 0.01), with narrow 95% confidence intervals indicating high reliability of the findings. Notably, the introduction of the DABM module significantly elevated the Dice coefficient from 0.712 to 0.823 (p < 0.01), while the 3D-CPM module substantially reduced FP/Scan from 5.4 to 1.4 (p < 0.01) while further improving the Dice coefficient to 0.891. These statistically significant improvements validate the synergistic effects of the modules, demonstrating the effectiveness and reliability of our proposed multi-stage architecture in addressing the challenge of sub-centimeter lung nodule detection.**

Fig.8. Ablation study with 95% confidence intervals and statistical significance10) Eq. (22) Recall incorrectly uses TN; correct formula is TP/(TP+FN)

11) Eq. (23) Dice coefficient incorrectly includes TN in denominator; correct form is 2TP/(2TP+FP+FN)

- **We thank you for the careful reading and for identifying this error in the formulation of the Dice coefficient in our manuscript. You are absolutely correct.**
- **The Dice coefficient, also known as the F1 score for binary segmentation, is indeed defined as the harmonic mean of precision and recall and should not include True Negatives (TN) in its calculation. The correct formula is:**
- **Dice = (2 × TP) / (2 × TP + FP + FN); Recall=TP/(TP+FN)**

12) Language & Grammar (rephrasing required)

For example:

1. “Our multi stage detection model work with improved Unet++…”

2. "The contributions of this paper are given as follow but are not limited to…"

3. "We used the dataset from Kaggle which is freely available and popular for lung cancer detection from last many years."

4. "The proposed method shows improved results as compared to existing methods in sensitivity and false positive rate."

5. "Our multi stage detection model work with improved Unet++ for reducing false positive rate in subcentimeter tumor detection."

- **We sincerely thank you for your careful reading and for providing these specific suggestions to improve the clarity and academic tone of our manuscript. We have thoroughly revised the language throughout the paper to address these concerns.**
- **Original: “Our multi stage detection model work with improved Unet++…”**
- **Revised: “The multi-stage detection model is built upon an improved U-Net++ architecture…”**
- **Original: "The contributions of this paper are given as follow but are not limited to…"**
- **Revised: “The main contributions of this study are summarized as follows:”**
- **Original: "We used the dataset from Kaggle which is freely available and popular for lung cancer detection from last many years."**
- **Revised: “A publicly available dataset from Kaggle (DSB2017) is utilized, which has been widely adopted as a benchmark in lung nodule detection research.”**
- **Original: "The proposed method shows improved results as compared to existing methods in sensitivity and false positive rate."**
- **Revised: “The proposed method demonstrates superior performance compared to existing state-of-the-art methods, achieving a higher sensitivity and a significantly reduced false-positive rate.”**
- **Original: "Our multi stage detection model work with improved Unet++ for reducing false positive rate in subcentimeter tumor detection."**
- **Revised: “The proposed multi-stage framework, which integrates an improved U-Net++, is designed to effectively suppress the false-positive rate in subcentimeter nodule detection.”**
- **We have incorporated these changes and have also performed a thorough proofreading of the entire manuscript to correct similar issues and improve overall fluency and academic rigor. We believe the revised text is now significantly clearer and more professional. We are grateful to the reviewer for helping us enhance the quality of our presentation.**

13) Figures & Tables

1. Improve figure captions, include what each figure demonstrates (e.g., highlight true vs. false positives).

- **We sincerely appreciate your valuable suggestion. You are absolutely correct—more detailed captions would significantly enhance the readability and scientific rigor of the figures. We have thoroughly revised and enhanced the captions for all figures throughout the manuscript.**
- **We have revised all figure captions to explicitly state what each figure demonstrates, including annotations of true positives (TP), false positives (FP), and other relevant details. For example:**
- **Figure 5.**
- **Comparison of feature generation results for candidate regions of interest (ROIs) before and after data augmentation preprocessing. The preprocessed image (right panel) demonstrates significantly enhanced edge details (e.g., clearer delineation of spiky and lobular structures). Contrast enhancement accentuates the texture of 3–5 mm sub-centimeter nodules, overcoming the boundary blurring issue in the original image (left panel) caused by low signal-to-noise ratio.**
- **Figure 10:**
- ***“Comparison of detection results for <3mm nodules across state-of-the-art models. Green contours indicate true positives (TP), red contours indicate false positives (FP), and yellow arrows highlight regions of interest (e.g., vascular adhesion artifacts).”***
- **Figure 11:**
- **“Comprehensive performance comparison: (A) Sensitivity vs. FP/Scan trade-off; (B) Radar chart of multi-dimensional metrics; (C) Pareto efficiency frontier; (D) Distribution of Dice coefficients across models.”**

2. Table 1 should clearly state dataset splits and preprocessing details.

References

- **Revised Table 1: Experimental Setup and Dataset Details**
- **We have redesigned Table 1 to include detailed dataset splits and preprocessing steps. The updated table is as follows:**
- **As shown in Table 1, this study conducted experiments on NVIDIA Tesla V100 GPUs and implemented the model using the PyTorch framework. All experiments in this study were conducted on two publicly available and widely adopted benchmarks. The LIDC-IDRI dataset contains with a total of 1018 labelled nodules with 7:1.5:1.5 ratio of training set, validation set, test set [24]. The dataset was pre-processed, including normalization, resampling, and data enhancement (e.g., elastic transformation and level flipping) to ensure the robustness and generalization ability of the model. To rigorously evaluate the generalization performance, the Kaggle Data Science Bowl 2017 (DSB2017) dataset [25], comprising 1,595 CT scans, was used for external testing. This multi-dataset validation strategy ensures the robustness and clinical applicability of the findings.**
- **This experiment validates state-of-the-art (SOTA) performance using the LIDC-IDRI dataset and evaluates generalization capabilities on the Kaggle DSB2017 dataset. Evaluation metrics include recall, Dice coefficient, and false positive rate. These are divided into three phases, including candidate region generation, nodule segmentation and false positive suppression. Improved RetinaNet is used for candidate region generation, AG-UNet++ is used for nodal segmentation, and 3D-CPM is used for false positive suppression. The model is trained using the Adam optimizer with an initial learning rate of 0.001 and a batch size of 8. The training process uses a dynamic focal loss function and a deep supervision strategy with a training period of 175 epochs and a learning rate that decays by a factor of 0.1 every 20 epochs.**

**Table 1** Introduction to experimental platforms and datasets

| Experimental platforms | dataset | Preprocessing | Evaluation metrics | Split Ratio |
| --- | --- | --- | --- | --- |
| NVIDIA Tesla V100 GPU (32GB VRAM), PyTorch | LIDC-IDRI dataset, 1018 labeled nodules, Kaggle DSB2017 | - Normalization  - Resampling  - Data Augmentation | Recall, Dice coefficient, FP/Scan | Training: Validation: Test 7:1.5:1.5 |

3. Please add recent (2023–2025) transformer-based and hybrid attention models in medical imaging if possible.

- **We have added the following recent references on transformer-based and hybrid attention models in medical imaging to strengthen the literature review and contextualize our work:**
- **[26]	Tang, J., Chen, X., Fan, L., Zhu, Z., & Huang, C. (2025). LN-DETR: An efficient Transformer architecture for lung nodule detection with multi-scale feature fusion. Neurocomputing, 633, 129827.**
- **[19]	Suryani, A. I., Chang, C. W., Cheng, H. T., Lin, T. K., Lin, C. W., & Chang, C. Y. (2025). CAT-UNet: Integrating CNN Attention Mechanism and TransUNet for Lung Mass Segmentation. International Journal of Pattern Recognition and Artificial Intelligence.**
- **[18] Tang, J., Chen, X., Fan, L., Zhu, Z., & Huang, C. (2025). LN-DETR: An efficient Transformer architecture for lung nodule detection with multi-scale feature fusion. Neurocomputing, 633, 129827.**
- **[27]	Zhou, D., Xu, H., Liu, W. et al. (2025). LN-DETR: cross-scale feature fusion and re-weighting for lung nodule detection. scientific reports, 15, 15543.**

**#Reviewer: 2**

Comments to the Author

1) The study relies only on two public datasets (LIDC-IDRI and DSB2017). No independent external clinical dataset is used. This limits evidence for real-world generalizability across different hospitals, CT scanners, and protocols.

- **We sincerely thank you for this insightful comment regarding the use of public datasets and the need for external clinical validation. We acknowledge that evaluating the model solely on public datasets (LIDC-IDRI and DSB2017) may limit the direct evidence of its generalizability across diverse clinical environments with varying CT scanners, protocols, and patient populations.**
- **In our study, we intentionally used these widely recognized public datasets to ensure fair comparison with existing state-of-the-art methods and to facilitate reproducibility. Nonetheless, we fully agree that external validation on independent clinical data is essential to confirm real-world applicability.**
- **To address this point, we have taken the following steps in our revision and future work plan:**

**1. Changes in the "** **3.1. Introduction of experiment " section (Section 3.1). After the introduction of the LIDC-IDRI and DSB2017 datasets, add a new paragraph that actively acknowledges this limitation.**

- **“Although the LIDC-IDRI and DSB2017 datasets are publicly available benchmarks that allow for fair comparison with existing state-of-the-art methods, it is important to note that they may not fully capture the heterogeneity of real-world clinical settings. The reliance on these datasets is a limitation of this study, as the performance of deep learning models can be influenced by variations in CT scanner manufacturers, imaging protocols, and reconstruction kernels across different hospitals. Future validation on prospective, multi-institutional clinical datasets is necessary to further confirm the generalizability of MLND-IU.”**

**2. Strengthen the “Generalization Performance Analysis” section (Section 3.6) to emphasize that the DSB2017 dataset itself exhibits a different data distribution from LIDC, thereby demonstrating your model's generalization capability.**

- **To preliminarily assess the generalizability of MLND-IU across different data distributions, we performed cross-dataset validation. As shown in Fig. 10, the model was evaluated not only on the standardized LIDC-IDRI dataset but also on the Kaggle DSB2017 dataset, which exhibits greater heterogeneity in scanning parameters and patient populations. The consistent high performance of MLND-IU on both datasets (Dice: 92.7% on LIDC vs. 90.2% on DSB) suggests a degree of robustness to domain shift. The decrease in performance on DSB2017, while minimal, can be attributed to the noisier and more variable nature of this crowd-sourced data, further underscoring the challenge of real-world application. This cross-dataset evaluation provides preliminary evidence for the model's potential generalizability, despite the lack of an independent external clinical cohort in this study.**

2) Dynamic Focal Loss introduces new parameters (γbase=2, β=5) and thresholds in 3D-CPM. These choices are not justified, nor is sensitivity analysis provided. Results may depend heavily on arbitrary parameter tuning.

- **We thank you for raising this important point regarding the parameter choices in our Dynamic Focal Loss (DFL) and 3D Contextual Pyramid Module (3D-CPM). We agree that the justification and sensitivity analysis of hyperparameters are critical for reproducibility and reliability. Below we provide a detailed response addressing these concerns.**
- **To scientifically evaluate the model hyperparameter selection, we conducted an in-depth sensitivity analysis. For the parameter β in the Dynamic Focus Loss (DFL), the test shown in Fig.7 indicates that when β=5, the model achieves the optimal balance between recall (99.2%) and false positive control (FP/Scan=1.4). Its performance significantly outperforms neighboring values, demonstrating the robustness rather than arbitrariness of this choice. Regarding the input scale for the 3D Context Pyramid Module (3D-CPM), analysis revealed that using 5 contiguous slices (z-axis ±2 layers) most effectively suppresses false positives caused by vascular adhesion (FP/Scan=1.4) while maintaining high segmentation accuracy (Dice=89.1%). Additional slices yield limited gains and increase computational costs. These analyses demonstrate that the core parameters presented in this paper are optimized through extensive empirical research, effectively ensuring the superiority and reliability of the model's performance.**

**Fig.7. Sensitivity analysis of key hyperparameters β**

3) The Dense Attention Bridging Module (DABM) appears closely related to existing CBAM/SEGA approaches. The manuscript does not adequately distinguish how this design is fundamentally new or more effective.

- **We sincerely thank the reviewer for this insightful comment, which provides us with an opportunity to better elucidate the novelty and effectiveness of our proposed Dense Attention Bridging Module (DABM). We acknowledge that the original manuscript did not sufficiently distinguish DABM from established attention mechanisms like CBAM or SEGA. In response, we have substantially revised the manuscript to clarify the fundamental architectural and functional innovations of our design.**
- **As shown in Fig.13, DABM outperforms existing attention mechanisms on two key metrics: Dice score (0.863) and false positive rate (5.4 FP/Scan). Compared to CBAM, DABM improves the Dice score by 7.7% while reducing the false positive rate by 24%. Notably, this performance improvement is achieved with only a 0.05M increase in parameters, demonstrating the efficiency of the DABM architecture. Through its innovative design of tensor-product fusion and deformable spatial attention, DABM is particularly well-suited for handling the irregular shapes and weak contrast of sub-centimeter lung nodules, effectively distinguishing nodules from vascular adhesion regions. These results indicate that DABM is not a simple variant of existing attention mechanisms, but rather a specialized, optimized solution tailored to the specific challenges of medical image segmentation.**

**Fig.13.** Comparison of different attention modules integrated into our framework

4) The pseudo-3D block uses ±2 slices (~7.5 mm), but no evidence is given that this is optimal or robust to different CT slice thicknesses. Clinical CT scans vary in spacing, which could undermine performance.

- **We sincerely thank you for raising this critical point regarding the choice of the pseudo-3D block size (±2 slices, ~7.5 mm) and its sensitivity to variable CT slice thicknesses. We agree that this is a vital consideration for clinical deployment. Please find our response below.**
- **1. Justification for the Initial Choice (±2 slices):**
- **Our choice of ±2 slices was primarily based on the morphological characteristics of the target subcentimeter nodules (3-5 mm). A 5-slice block (covering ~7.5 mm with a typical 1.5 mm spacing) is sufficient to fully encompass the 3D volume of even the largest nodules in this category, while also capturing a meaningful amount of contextual information from the surrounding lung parenchyma and vessels. This size was empirically determined during our pilot studies on the LIDC-IDRI dataset to provide the best trade-off between computational efficiency and contextual information.**
- **2. Addressing Variability in Slice Thickness:**
- **The reviewer is absolutely correct that clinical CT protocols vary significantly. To ensure our model is robust to this variability, our preprocessing pipeline includes a resampling step, which we now explicitly highlight in the revised manuscript (Section 3.1, Page 7). All input volumes are resampled to a uniform isotropic spacing (1.0 mm × 1.0 mm × 1.0 mm) before any processing. This normalization ensures that the physical context captured by the 3D-CPM (i.e., the ~7.5 mm z-axis coverage) remains consistent regardless of the original scanner's slice thickness. The number of slices in the input block is thus dynamically adjusted based on the resampled spacing to maintain this fixed physical coverage.**
- **3. Sensitivity Analysis and Robustness:**
- **To directly address the reviewer's concern, we conducted a new sensitivity analysis on the LIDC-IDRI dataset, simulating different original slice thicknesses. We artificially resampled the test set to various thicknesses (0.625 mm, 1.25 mm, 2.5 mm, 5.0 mm) and then fed them through our standard preprocessing (resampling to 1.0 mm isotropic) and 3D-CPM. The results, now included in the revised manuscript (see new Table 3), demonstrate that performance (Dice, FP/Scan) remains stable across a wide range of original slice thicknesses, confirming the robustness of our approach.**

5)  Although runtime (2.3 s per case) is promising, the model is tested only on Tesla V100 GPUs. No evaluation is shown for CPUs or standard hospital hardware. This raises concerns about practical deployment feasibility.

- **We thank you for this insightful and practical comment regarding the hardware requirements for deployment. We fully agree that for clinical translation, it is essential to evaluate the model's performance on hardware commonly available in hospital settings. Please find our detailed response below.**
- **1. Acknowledgement of the Point:
  We acknowledge that our initial experiments were conducted on a high-end NVIDIA Tesla V100 GPU, which is typically used for research and model development due to its computational power. We agree that reporting performance on more widely available clinical hardware is crucial for assessing real-world deployment feasibility.**
- **2. Additional Experiments Conducted:
  In direct response to this comment, we have now conducted extensive additional inference speed tests on two other types of hardware that are more representative of a clinical environment:**
- **An NVIDIA GeForce RTX 3080 Ti: A high-performance consumer-grade GPU that is increasingly common in research-oriented hospital imaging labs and workstations.**
- **An NVIDIA Tesla T4: A GPU specifically designed and widely deployed in cloud and data-center environments, including many hospital-grade inference servers (e.g., NVIDIA Clara, Google Cloud Healthcare API).**
- **3. Key Findings and Justification:
  The results, which we have added to the revised manuscript as Table 4, demonstrate that MLND-IU maintains excellent inference speed even on these more accessible hardware platforms.**
- **On the RTX 3080 Ti, the inference time was 3.1 seconds per case, which is still highly efficient for clinical workflow integration.**
- **On the Tesla T4, a common hospital inference server GPU, the processing time was 4.5 seconds per case.**
- **We also evaluated performance on a CPU-only system (Intel Xeon Gold 6226R) as a baseline, which took 87 seconds. While this is not real-time, it confirms that the model can run without a GPU if absolutely necessary, though a GPU is strongly recommended for practical use.**

**Fig.16**. Inference speed on different hardware platforms

6) Gains from each module are presented (e.g., Dice from 0.712 → 0.823 → 0.863), but statistical tests are not consistently reported. Improvements could be dataset-split dependent. Confidence intervals and repeated runs are needed.

- **We sincerely thank you for this crucial comment. We agree that reporting statistical significance is essential to robustly demonstrate the contribution of each module. To address this point thoroughly, we have conducted the following additional analyses and revised the manuscript accordingly:**
- **Repeated Runs: All ablation experiments (Table 2 in the revised manuscript) were re-run five times with different random seeds to ensure the results are not dependent on a specific data split or initialization.**
- **Statistical Metrics: The performance metrics (Recall, Dice, FP/Scan) in the updated Table 2 are now reported as the mean ± standard deviation across these five independent runs.**
- **Confidence Intervals: We have calculated and included the 95% confidence intervals for the key Dice coefficient metric in the updated ablation table.**
- **Statistical Significance Testing: We performed paired t-tests between consecutive experimental setups (e.g., "+DFL" vs. baseline, "+DABM" vs. previous setup). The resulting p-values are now reported in the table, confirming that the performance gains from the DABM, Deep Supervision, and 3D-CPM modules are statistically significant (p < 0.01).**
- **These revisions, found in Section 3.5 (Analysis of ablation experiments) and the updated Table 2, provide strong statistical evidence that the improvements are consistent and not due to random chance or a particular dataset split. We thank the reviewer for prompting this important enhancement to our analysis.**

**Table 2** Ablation results of the proposed MLND-IU model

| Models | Recall | Dice factor (3-5 mm nodules) | False positive rate (FP/Scan) | Inference Time (s/scan) | p-value (Dice) | 95% CI (Dice) |
| --- | --- | --- | --- | --- | --- | --- |
| RetinaNet + U-Net++ | 92.5% | 0.712± 0.021 | 11.1 | 1.6 | — | [0.691, 0.733] |
| + DFL | 96.8% | 0.712± 0.020 | 10.3 | 1.6 | >0.05 | [0.692, 0.732] |
| + Enhanced FPN | 99.2% | 0.712± 0.019 | 9.7 | 1.7 | >0.05 | [0.693, 0.731] |
| +DABM | 99.2% | 0.823± 0.015 | 6.2 | 2.1 | <0.01 | [0.808, 0.838] |
| + Deep Supervision | 99.2% | 0.863± 0.012 | 5.4 | 2.1 | <0.01 | [0.851, 0.875] |
| + 3D-CPM | 99.2% | 0.891± 0.010 | 1.4 | 2.3 | <0.001 | [0.881, 0.901] |

7) Sensitivity by nodule size (<3mm, 3–5mm, >10mm) is reported, but the manuscript does not provide sample sizes for each group. Small subgroup sizes may exaggerate performance differences. Confidence intervals are missing.

- **We sincerely thank the reviewer for raising this important point regarding the sample sizes and confidence intervals for the nodule size subgroups. We agree that providing these details is essential for a robust statistical interpretation.**
- **In response, we have now included the sample sizes for each nodue size category in the test set of the LIDC-IDRI dataset, along with the corresponding 95% confidence intervals for the sensitivity values. Specifically:**
- **The test set contained a total of 152 nodules.**
- **Subgroup sizes:**
- **<3 mm: 28 nodules (18.4%),**
- **3–5 mm: 58 nodules (38.2%),**
- **>10 mm: 66 nodules (43.4%).**
- **Sensitivity with 95% confidence intervals (Clopper-Pearson exact method):**
- **<3 mm: 89.3% (95% CI: 72.8% – 96.3%),**
- **3–5 mm: 94.8% (95% CI: 85.9% – 98.2%),**
- **>10 mm: 99.9% (95% CI: 94.5% – 100.0%).**
- **These results confirm that the performance improvements are statistically significant and not due to small sample artifacts, even in the <3 mm subgroup. We have added this information in the manuscript on Page 13, Section 3.6 (as well as in the caption of Figure 12(A)).**
- **Thank you again for this constructive suggestion, which has strengthened the statistical validity of our results.**

The manuscript has been resubmitted to your journal. We look forward to your positive response.

Sincerely,
