# Supplementary material for: MLND-IU: A multi-stage detection model of subcentimeter lung nodule with improved U-Net++
Source: PLoS One. 2026 Feb 6;21(2):e0341750. doi: 10.1371/journal.pone.0341750 (PMC12880664; doi:10.1371/journal.pone.0341750)
Supplement: Attachment — Submitted filename: 1PONE-D-25-41911_point_by_point_auresp_2.docx [file pone.0341750.s005.docx]

< ***PLOS ONE***>

<**PONE-D-25-41911 R1**>

**#** **Editor**

Thank you for submitting your manuscript to PLOS ONE. After careful consideration, we feel that it has merit but does not fully meet PLOS ONE’s publication criteria as it currently stands. Therefore, we invite you to submit a revised version of the manuscript that addresses the points raised during the review process.

Please submit your revised manuscript by Nov 23 2025 11:59PM. If you will need more time than this to complete your revisions, please reply to this message or contact the journal office at [plosone@plos.org](mailto:plosone@plos.org). When you're ready to submit your revision, log on to https://www.editorialmanager.com/pone/ and select the 'Submissions Needing Revision' folder to locate your manuscript file.

Please include the following items when submitting your revised manuscript:

- A rebuttal letter that responds to each point raised by the academic editor and reviewer(s). You should upload this letter as a separate file labeled 'Response to Reviewers'.
- A marked-up copy of your manuscript that highlights changes made to the original version. You should upload this as a separate file labeled 'Revised Manuscript with Track Changes'.
- An unmarked version of your revised paper without tracked changes. You should upload this as a separate file labeled 'Manuscript'.

If you would like to make changes to your financial disclosure, please include your updated statement in your cover letter. Guidelines for resubmitting your figure files are available below the reviewer comments at the end of this letter.

**Dear Editor,**

**Thank you very much for handling our manuscript and for providing the constructive comments and suggestions from the reviewers. We also extend our sincere gratitude to the reviewers for their thorough and insightful feedback, which has greatly helped us to improve the quality of our work.**

**We have carefully considered all the points raised and have revised the manuscript accordingly. Below, we provide a point-by-point response to the comments from the Reviewers 1 and 2. All changes in the revised manuscript have been highlighted using the “Track Changes” feature.**

**Thank you once again for your time and consideration.**

**Best regards,**

**#Reviewer: 1**

Comments to the Author
The authors have successfully responded to most of the issues. The quality of the manuscript has been significantly improved, with unnecessary text removed and contributions clearly stated. All major technical gaps including datasets, metrics, baselines, and significance testing have now been addressed. The work convincingly demonstrates computational efficiency and clinical viability, and the language and presentation have been refined. In addition, the figure captions have been corrected, further enhancing the clarity of the manuscript.

However, formatting is still not up to the mark, no spacing between text and figures, figure 3 text looks like a screenshot.

1) Few sentences are floating in the paper "This operation is used to extract localized morphological features (e.g., burrs, foliations) of the nodule while suppressing noise"

2) Unnecessary use of caps "Dynamic Focus Loss Function"

3) In entire manuscript if short or abbreviated form acronyms is defined it should be used not the full form. e.g. context pyramid module (3D-CPM), no consistency sometimes its with small letters or viceversa.

4) paragraph indentation is following no consistency look at page 7.

- **We sincerely thank you for your thorough review and positive feedback on the improvements made to our manuscript. We are pleased that the clarifications and enhancements have strengthened the paper. Regarding the remaining formatting and presentation points you raised, we have addressed them as follows:**
- **1. Regarding floating sentences and formatting: We have carefully reviewed the entire manuscript to ensure proper integration of text and figures. The specific sentence you mentioned, along with other similar instances, has been integrated into the main paragraph flow. All figures and text now have appropriate spacing.**
- **2. Regarding capitalization: We have standardized the capitalization throughout the manuscript. Terms like "Dynamic Focal Loss function" now consistently use standard capitalization rules, avoiding unnecessary capitalization.**
- **3. Regarding acronym consistency: We have rigorously checked the entire manuscript for acronym usage. After an acronym is defined (e.g., 3D Contextual Pyramid Module (3D-CPM)), the abbreviated form (3D-CPM) is used consistently thereafter. The formatting of acronyms has also been standardized.**
- **4. Regarding paragraph indentation: We have applied consistent paragraph indentation formatting across the entire manuscript, including on page 7, to improve visual presentation and readability.**
- **Furthermore, we have replaced the low-resolution text in Figure 3 with high-quality, editable text to ensure clarity.**

**#Reviewer: 2**

Comments to the Author

1) A final round of proofreading will help polish the manuscript.

2) Some figure and table captions remain too brief. Captions should clearly explain what the figure shows, what each color or label represents, and define all abbreviations so that the figure is understandable without referring to the main text.

3) specify the sample size (n) used in each statistical comparison to strengthen transparency and reproducibility.

- **We thank you for your valuable comments aimed at further polishing the manuscript. We have implemented the following changes:**
- **1. Final Proofreading: The manuscript has undergone a final round of careful proofreading by the authors to correct any remaining grammatical errors, improve sentence fluency, and enhance overall language quality.**
- **2. Enhanced Figure and Table Captions: We have significantly expanded all figure and table captions to make them self-contained. The revised captions now clearly describe what each figure/table shows, explain the meaning of colors, labels, symbols, and define all abbreviations used within the figure/table itself. This ensures they are fully understandable without requiring reference to the main text.**
- **3. Specification of Sample Sizes: In the revised manuscript, we have explicitly stated the sample size (n) for each statistical comparison reported in the Results section, particularly in the ablation studies and performance comparisons. This enhances the transparency and reproducibility of our findings.**
- **Thank you again for your insightful comments, which have undoubtedly helped us improve the quality and clarity of our work.**

The manuscript has been resubmitted to your journal. We look forward to your positive response.

Sincerely,
