# Supplementary material for: MLND-IU: A multi-stage detection model of subcentimeter lung nodule with improved U-Net++
Source: PLoS One. 2026 Feb 6;21(2):e0341750. doi: 10.1371/journal.pone.0341750 (PMC12880664; doi:10.1371/journal.pone.0341750)
Supplement: Attachment — Submitted filename: PONE-D-25-41911R2-Fatima (5).docx [file pone.0341750.s004.docx]

In abstract, P-values are used without context (e.g., what test was used, which comparison group). State statistical validation methods and dataset independence more transparently.

The introduction reads like a mini-survey; many cited works (e.g., [10]–[23]) are summarized without synthesis. The paper does not specify how much existing methods fail in subcentimeter cases (e.g., exact FP/Scan thresholds from literature). Condense the literature into 3–4 thematic groups (imbalance, attention, context modeling, 3D fusion). Clearly articulate what specific deficiency each stage addresses and why a cascade is necessary.

Missing kernel sizes, feature map dimensions, layer counts, and parameter distribution per block. No training/inference diagram showing how outputs of each stage connect. Why not 2 or 4 stages? Replace long derivations with one composite architecture diagram showing data flow and tensor sizes. Provide a configuration table summarizing: backbone, input size, loss per stage, optimizer, and memory footprint. Clarify whether the three stages are jointly trained or trained sequentially (text implies sequential fine-tuning, but not stated).

No comparison to other adaptive loss variants (e.g., ASL, GHM, Focal Tversky). Parameters (β=5, γ_base=2) are empirically fixed but no cross-validation justification.Add an empirical comparison table with other imbalance-focused losses. Visualize gradient modulation vs. p_i to support claims about stability.

No ablation isolating the benefit of this module separately from DFL. Missing FLOPs impact and convergence speed analysis. Cross-scale weights (W_lk) are introduced but not explained: are they shared across channels or per-feature? Provide parameter overhead and runtime cost. Add heatmaps showing how the module amplifies subcentimeter nodules in feature space.

DABM efficiency claim (only +0.05M params) is unsupported by architectural detail. No boundary visualization to justify “edge enhancement”. Show feature activation maps before and after DABM. Compare to existing attention modules (CBAM, SE, ECA, Criss-Cross) quantitatively and visually. Clarify whether DABM weights are shared across layers.

Include a visual example of vessel-adhesion suppression. Conduct an ablation comparing slice contexts: ±1, ±2, ±3. Quantify memory vs. accuracy trade-off.

Perform 5-fold cross-validation on LIDC-IDRI. Add external validation using an independent institutional dataset. Explicitly follow LUNA16 challenge definitions for FP/Scan and sensitivity. Include boxplots of Dice across cases to illustrate robustness. Standardize reporting: use FROC-AUC and 95% CI for all major metrics.

No measure of computational cost per ablation (FLOPs or latency per module). n=152 samples is too small for multi-parameter inference; p-values may be inflated. Re-run ablations with randomized 3 splits to confirm consistency. Include per-stage latency and FLOPs per scan. Add qualitative examples illustrating visual differences between each ablation.

Comparisons are not standardized: different input resolutions, patch sizes, or preprocessing pipelines are likely. “AUC=0.964” contradicts the earlier “AUC=0.84” in abstract, unclear if this refers to detection or malignancy classification. Runtime comparison lacks reproducibility (hardware details differ between models). Claim that MLND-IU “defines the theoretical clinical limit” is scientifically unjustified. Re-evaluate baselines using identical preprocessing, patch sizes, and inference hardware. Clearly separate detection AUC vs. malignancy AUC.

DSB2017 is not truly heterogeneous; no cross-institutional or low-dose datasets used. No assessment of robustness to noise, slice thickness variability, or domain shift. Inference times (2.3–4.5 s/case) are promising but do not include preprocessing and postprocessing latency. Test on non-public multicenter or low-dose datasets. Include robustness studies (noise, different kernels, contrast-enhanced scans). Report total pipeline latency (from DICOM input to final mask).

Reword claims conservatively: “demonstrates substantial improvement…” instead of “redefines.” Add future directions: interpretability (Grad-CAM, SHAP), uncertainty modeling, and clinical reader studies. Discuss ethical implications and reproducibility (e.g., release anonymized model weights, code, and split lists).

Please refer to

Abbas, Waseem, et al. "Lungs nodule cancer detection using statistical techniques." 2020 IEEE 23rd International multitopic conference (INMIC). IEEE, 2020.

Awan, Tehreem, and Khan Bahadar Khan. "Analysis of underfitting and overfitting in u-net semantic segmentation for lung nodule identification from x-ray radiographs." 2023 IEEE International Conference on Emerging Trends in Engineering, Sciences and Technology (ICES&T). IEEE, 2023.

Awan, Tehreem, and Khan Bahadar Khan. "Investigating the impact of novel XRayGAN in feature extraction for thoracic disease detection in chest radiographs: lung cancer." Signal, Image and Video Processing 18.5 (2024): 3957-3972.

Awan, T., Khan, K. B., & Mannan, A. (2023). A compact CNN model for automated detection of COVID-19 using thorax x-ray images. Journal of Intelligent & Fuzzy Systems, 44(5), 7887-7907.
