# Supplementary material for: MLND-IU: A multi-stage detection model of subcentimeter lung nodule with improved U-Net++
Source: PLoS One. 2026 Feb 6;21(2):e0341750. doi: 10.1371/journal.pone.0341750 (PMC12880664; doi:10.1371/journal.pone.0341750)
Supplement: Attachment — Submitted filename: PONE-D-25-41911R4_point_by_point.docx [file pone.0341750.s008.docx]

< ***PLOS ONE***>

<**PONE-D-25-41911R4**>

**#** **Academic Editor**

Thank you for submitting your manuscript to PLOS ONE. After careful consideration, we feel that it has merit but does not fully meet PLOS ONE’s publication criteria as it currently stands. Therefore, we invite you to submit a revised version of the manuscript that addresses the points raised during the review process.

**Dear Khan Bahadar Khan, Ph.D,**

**Thank you very much for handling our manuscript and for providing the constructive comments and suggestions from the reviewers. We also extend our sincere gratitude to the reviewers for their thorough and insightful feedback, which has greatly helped us to improve the quality of our work.**

**We have carefully considered all the points raised and have revised the manuscript accordingly. Below, we provide a point-by-point response to the comments from the Reviewers. All changes in the revised manuscript have been highlighted using the “Track Changes” feature.**

**Thank you once again for your time and consideration.**

**Best regards,**

**#Reviewer: 1**

1）Authors have significantly improved the quality of the manuscript but providing more details and improving the shortcomings as highlighted by the reviewers, however the presentation especially the figure quality text, labels are very poorly shown.

- **Thank you for highlighting the issue with the figure quality. We agree that clear, high-resolution figures are essential. In the revised manuscript all figures have been carefully updated to improve text legibility and label clarity.**
- **In the revised manuscript, we have comprehensively improved the quality and readability of all figures. Specifically:**
- **1. All figures have been regenerated at high resolution (≥300 dpi) to ensure clarity in both online and print formats;**
- **2. Text, labels, legends, and annotations in all figures have been enlarged and standardized to improve legibility;**
- **3. Font styles and sizes have been unified across figures to maintain visual consistency;**
- **4. Several complex framework and result figures (e.g., the overall MLND-IU architecture, ablation analysis, and performance comparison plots) have been carefully redrawn to avoid overcrowding and overlapping elements;**
- **5. Figure captions have been revised to provide clearer and more detailed explanations, facilitating independent interpretation without relying excessively on the main text.**
- **We believe these revisions have substantially enhanced the visual quality and interpretability of the manuscript, and have effectively addressed the reviewer’s concerns regarding figure presentation.**

**#Reviewer: 3**

1) Clearly identify one or two key unresolved gaps in existing methods and align the proposed architecture directly to those gaps.

- **We appreciate your insightful comment. Following this suggestion, we have explicitly identified two critical unresolved gaps in existing lung nodule detection methods and have revised the manuscript to directly align each component of the proposed MLND-IU architecture with these gaps.**
- **(1) Unresolved Gap 1: Insufficient sensitivity for subcentimeter nodules under extreme class imbalance**
- **Most existing detection frameworks suffer from degraded sensitivity for subcentimeter nodules (<5 mm) due to severe foreground–background imbalance and feature dilution in deep networks. While attention mechanisms and Transformers have been introduced, they typically rely on static weighting schemes and do not adaptively emphasize hard samples, resulting in missed detections of tiny nodules.**
- **Revision and alignment:**

**This gap is now explicitly articulated in the Introduction.**

- **To address it, we align the first-stage architecture of MLND-IU with this challenge by introducing Dynamic Focal Loss (DFL) and a cross-scale enhanced FPN, which jointly adapt gradient weighting based on sample difficulty and reinforce low-level feature responses for tiny nodules.**
- **The architectural alignment between this gap and the first-stage design is now clearly summarized in Fig. 1 (overall framework) and elaborated in Section 2.2.**
- **(2) Unresolved Gap 2: Inadequate suppression of vascular-adhesion false positives caused by limited 3D contextual modeling**
- **Many existing approaches rely primarily on 2D slice-based analysis or weak 3D aggregation, which fails to exploit spatial continuity along the z-axis. As a result, vascular adhesions and bronchial structures that resemble nodules remain a major source of false positives, particularly in subcentimeter cases.**
- **Revision and alignment:**
- **This limitation has been clearly emphasized in the revised Introduction.**
- **The third-stage 3D Contextual Pyramid Module (3D-CPM) is now explicitly presented as a targeted solution to this gap, leveraging multi-slice contextual modeling to differentiate isolated nodules from continuous vascular structures.**
- **Detailed architectural justification and mathematical formulation have been added in Section 2.4, with corresponding visual clarification in Fig. 4 (3D-CPM module).**
- **Quantitative evidence linking this design choice to false-positive suppression has been strengthened in Section 3.5 (Ablation Study, Table 2), demonstrating an 87.3% reduction in FP/Scan after introducing 3D-CPM.**
- **We believe these revisions directly address your concern and clearly demonstrate how the proposed architecture is purposefully designed to resolve the most critical limitations of existing methods.**

2) Several components (dynamic loss weighting, attention-guided skip connections, pseudo-3D context) resemble known techniques with incremental modifications. The manuscript does not sufficiently distinguish what is fundamentally new versus what is adapted.

- **We thank the reviewer for this important observation. We fully agree that it is essential to clearly distinguish between adapted techniques and the fundamentally new contributions of this work. In response, we have revised the manuscript to explicitly clarify the novelty of each component and its distinction from prior methods.**
- **(1) Dynamic loss weighting — distinction from conventional focal-loss-based methods**
- **While focal loss and its variants have been widely used to mitigate class imbalance, existing approaches typically employ fixed or heuristically tuned modulation factors that are insensitive to sample difficulty variations during training.**
- **Fundamental novelty:**
- **We propose a sample-adaptive Dynamic Focal Loss (DFL) in which the modulation factor is explicitly coupled to the calibrated prediction confidence, enabling continuous, difficulty-aware gradient reweighting rather than static suppression of easy samples.**
- **This design is specifically tailored to extreme imbalance scenarios in subcentimeter lung nodule detection, where positive samples account for less than 0.1% of voxels.**
- **(2) Attention-guided skip connections — distinction from existing attention mechanisms (e.g., SE, CBAM)**
- **Although attention-based skip connections have been explored in U-Net variants, most existing methods apply channel or spatial attention independently, or rely on static attention weights that are not optimized for small-target boundary enhancement.**
- **Fundamental novelty:**
- **The proposed Dense Attention Bridging Module (DABM) introduces a tensor-product fusion of channel attention and deformable spatial attention, enabling higher-order feature interactions rather than sequential or additive attention modulation.**
- **Embedding DABM densely within the nested skip connections of U-Net++ creates a structurally different attention propagation pattern that explicitly enhances edge-sensitive features of 3–5 mm nodules.**
- **(3) Pseudo-3D contextual modeling — distinction from conventional 2.5D or shallow 3D methods**
- **Pseudo-3D and multi-slice input strategies have been previously reported; however, many treat adjacent slices as simple channel stacking without explicit hierarchical context modeling.**
- **Fundamental novelty:**
- **The proposed 3D Contextual Pyramid Module (3D-CPM) is not a naive pseudo-3D extension but a multi-level context pyramid that jointly models local, regional, and global morphology along the z-axis, with adaptive fusion weights learned end-to-end.**
- **Additionally, the dynamic thresholding strategy explicitly leverages contextual response statistics to suppress vascular-adhesion false positives, which is absent in prior pseudo-3D approaches.**
- **We believe these revisions clearly delineate what is new in this work versus what builds upon prior research, and more accurately position the contribution of MLND-IU within the existing literature.**

3) Almost every comparison is reported as statistically significant, but effect sizes are not consistently discussed. With large sample sizes, statistical significance alone is insufficient.

- **We thank you for this valuable comment and fully agree that statistical significance alone may be misleading in large-scale datasets. To address this concern, we have systematically revised the manuscript to explicitly report and interpret effect sizes alongside p-values, thereby providing a more meaningful assessment of practical significance.**
- **Specific revisions are as follows:**
- **(1) Explicit reporting of effect sizes for major performance comparisons: For all key quantitative comparisons (sensitivity, specificity, F1-score, CPM, and FP/Scan), we have added corresponding effect size metrics, including Cohen’s d for continuous performance measures and relative percentage improvement for clinically interpretable metrics. These effect sizes are now consistently reported in Tables 1–3.**
- **(2) Integration of effect size interpretation into result analysis: The Results section has been revised to explicitly interpret the magnitude of observed improvements (small, moderate, or large effect), rather than relying solely on statistical significance. In particular, we now emphasize that although several improvements reach statistical significance, only those with moderate-to-large effect sizes are highlighted as clinically meaningful.**
- **(3) Subgroup and ablation analyses focusing on effect magnitude: For the subcentimeter nodule subgroup (<5 mm), we have added a dedicated discussion on effect size to demonstrate that performance gains are not only statistically significant but also substantively larger than those observed in larger nodules. Similarly, in the ablation study, we now report effect size changes introduced by each module (DFL, DABM, and 3D-CPM), clarifying their relative practical contributions.**
- **(4) Methodological clarification on statistical analysis: We have added a short paragraph clarifying the rationale for combining hypothesis testing with effect size reporting, and explicitly note the limitations of p-values in large-sample settings.**
- **To improve transparency, we have added a concluding sentence in the Results section emphasizing that claims of superiority are based on both statistical significance and effect magnitude, ensuring that the reported improvements reflect meaningful clinical relevance rather than sample-size-driven significance.**
- **We believe these revisions substantially strengthen the statistical rigor of the manuscript and directly address the reviewer’s concern.**

4) Almost all ablation results strongly favor the proposed modules, with minimal discussion of failure cases or trade-offs. This one-sided presentation reduces credibility.

- **We thank you for this valuable and constructive comment. We agree that a comprehensive evaluation should not only highlight performance gains but also discuss failure cases and practical trade-offs. To address this concern and improve the credibility and balance of the manuscript, we have substantially revised the experimental analysis and discussion as follows.**
- **(1) Added explicit discussion of failure cases: We have incorporated a dedicated subsection analyzing representative failure cases, focusing on scenarios where the proposed modules do not provide consistent benefits, such as extremely low-contrast nodules adjacent to dense vascular bundles and irregularly shaped nodules with ambiguous boundaries.**
- **(2) Analysis of performance trade-offs and computational overhead: While the proposed modules improve detection accuracy, they introduce additional parameters and computational cost. We now explicitly quantify these trade-offs.**
- **(3) Balanced interpretation of ablation results: We have revised the ablation discussion to explicitly acknowledge cases where performance gains are marginal or dataset-dependent, rather than uniformly dominant.**
- **We believe these additions provide a more transparent and credible evaluation of the proposed method, and directly address the reviewer’s concern regarding the presentation of ablation results.**

5) It is not always clear whether competing models were retrained under identical conditions or evaluated using reported results, which may bias comparisons.

- **We thank you for raising this important concern regarding the fairness and reproducibility of model comparisons. To eliminate any ambiguity and potential bias, we have revised the manuscript to explicitly clarify the evaluation protocol used for all competing methods. All competing models were reimplemented and retrained under identical experimental settings as MLND-IU, including the same data splits, preprocessing pipeline, training strategy, and hyperparameter configuration, ensuring a fair and unbiased comparison.**
- **Clarification of evaluation strategy:**
- **1. For methods with publicly available implementations, we retrained all competing models under identical experimental conditions, including the same training–validation–test splits, identical input resolution, data preprocessing pipeline, augmentation strategy, optimizer type, learning rate schedule, batch size, and training epochs.**
- **2. No hybrid setting (partial retraining combined with reported results) was used for any single method.**
- **We believe these revisions clearly resolve the reviewer’s concern and ensure that all comparative results presented in this study are fair, transparent, and reproducible.**

6）Improvements of 15–20% over strong baselines (e.g., nnU-Net) are unusually large and warrant more cautious interpretation and independent validation.

- **We appreciate your careful and constructive concern. We fully agree that performance gains of this magnitude over strong baselines such as nnU-Net require cautious interpretation and clear contextualization. In response, we have revised the manuscript to clarify the conditions under which these improvements occur, to moderate the interpretation of the results, and to strengthen independent validation.**
- **Clarification of performance gains: First, we clarify that the reported 15–20% improvements do not reflect overall detection performance across all nodules, but are observed under challenging and clinically critical conditions, particularly for subcentimeter nodules (<5 mm) and false-positive–sensitive operating points. Second, we have revised the manuscript to adopt a more cautious and balanced interpretation of the results. The revised text emphasizes that nnU-Net remains a strong and well-generalized baseline, while the proposed method demonstrates task-specific advantages under extreme class imbalance and tiny-object detection scenarios.**

The manuscript has been resubmitted to your journal. We look forward to your positive response.

Sincerely,
