# Supplementary material for: MLND-IU: A multi-stage detection model of subcentimeter lung nodule with improved U-Net++
Source: PLoS One. 2026 Feb 6;21(2):e0341750. doi: 10.1371/journal.pone.0341750 (PMC12880664; doi:10.1371/journal.pone.0341750)
Supplement: Attachment — Submitted filename: PONE-D-25-41911R4.docx [file pone.0341750.s007.docx]

All reviewer comments have been fully and satisfactorily addressed in the revised manuscript. Figure quality and presentation have been substantially improved through high-resolution regeneration, clearer labeling, and enhanced captions. The manuscript now clearly identifies key unresolved gaps in existing methods and explicitly aligns each component of the proposed architecture to those gaps, while clearly distinguishing novel contributions from adapted techniques. Statistical analyses have been strengthened by consistently reporting and interpreting effect sizes alongside significance testing, and the ablation study has been balanced with discussions of failure cases, trade-offs, and computational cost. The evaluation protocol has been clarified to ensure fair and reproducible comparisons, and large performance gains over strong baselines are now interpreted more cautiously and contextually. Overall, the revisions significantly enhance clarity, rigor, transparency, and credibility of the work.
